# A multicentric observational retrospective study on patients with short bowel syndrome and chronic intestinal failure who underwent intestinal transplantation in Brazil

**DOI:** 10.1016/j.clinsp.2024.100521

**Published:** 2024-10-25

**Authors:** Yuri Longato Boteon, Mariana Hollanda Martins da Rocha, Luciana Haddad, Rafael Antonio Arruda Pecora, Andre Dong Won Lee, Claudia Yang Santos, Amanda Pinter Carvalheiro da Silva Boteon, Igor Calil, Giovana Garcia Rossi, Fernanda Marques, Bianca Facas, Luiz Augusto Carneiro D'Albuquerque

**Affiliations:** a Hospital Israelita Allbert Einstein, São Paulo, SP, Brazil; b Hospital das Clínicas da Faculdade de Medicina da Universidade de São Paulo, São Paulo, SP, Brazil; c Takeda Distribuidora Ltda, São Paulo, SP, Brazil

**Keywords:** Health care costs, Short bowel syndrome, Intestinal transplantation

## Abstract

**Introduction:**

Short Bowel Syndrome (SBS) is a rare gastrointestinal disorder associated with Intestinal Failure (SBS‐IF) that leads to morbidity, mortality, and a burden on healthcare costs. Intestine Transplantation (IT) is a treatment option for patients with SBS-IF as it replaces the missing or diseased intestine and offers the potential for return to normal activities and intestinal function. This study aims to describe the clinical course and demographical and clinical characteristics of subjects with SBS-IF who underwent IT in Brazil.

**Methods:**

This retrospective observational study included all SBS-IF patients who underwent IT in two reference centers in Brazil from April 2011 to December 2021.

**Results:**

A total of 7 young male participants were included in the study. The most frequent underlying condition was surgical complications, followed by intestinal volvulus and incisional hernia. The most frequent indication for IT was a hepatic disease associated with total Parenteral Nutrition (PN). The main type of IT performed was intestine only. The median time from underlying condition to IT was 67.3 (16.5‒88.5) months. The mean (SD) number of yearly hospitalizations per patient was 0.5 (0.3). The most common reason for hospitalization was PN-related complications. Sixty exams were performed in-hospital and 53 in the outpatient setting.

**Conclusion:**

The findings of this study may be helpful to understand better the journey of patients with SBS-IF to IT in Brazil, providing real-world evidence to develop health policy guidelines and improve the quality of life of these patients.

## Highlights

• Substantial average of hospitalizations (4.3 per patient and 0.5 patient/year).
• The main reasons for intestine transplantation were related to parenteral nutrition.
• Complications related to parenteral nutrition were the main reason for hospitalization.
• High burden of hospitalization and complication with a mean length of stay of 50 days.
• Median time between SBS-IF diagnosis and transplant indication of 16 months.

## Introduction

Short Bowel Syndrome (SBS) is a rare condition caused by an extensive resection of the small bowel, leading to nutrient malabsorption [1]. This disease negatively impacts patients' quality of life and represents an important burden for healthcare systems worldwide [2]. The general prevalence of SBS ranges from 0.4 to 40 cases per million people in developed countries [1]. In Brazil, there is no accurate data on the frequency of SBS. Consequently, the occurrence of SBS is commonly estimated based on the proportion of patients on prolonged use of Parenteral Nutrition (PN).

SBS may progress to chronic Intestinal Failure (SBS-IF), a more severe condition indicated by a long-lasting reduction of gut mass or its function below the minimum necessary to absorb the required fluids and nutrients [3]. Usually, it is associated with a remnant small bowel length of <75 cm and with the loss of a large part of the original colon [1]. For this reason, these patients are dependent on PN for a few years or even for the rest of their life [1] to have adequate nutrient levels [4]. The PN dependency rate in patients with SBS may vary from 75 % to 50 % at 1 and 5 years from the beginning of the SBS condition, respectively.

Patients with SBS-IF may present several complications, such as uncontrolled diarrhea, intestinal failure-associated liver disease, and cholelithiasis, among others; besides the complications related to the long-term PN use, including bacterial infections, blood clots, iron deficiency anemia, central venous thrombosis, hepatic injury, and others [2],[5]. Studies estimate that around 1 to 3 persons per million of the general population will present IF annually. Of those, 15 % will be candidates for intestinal or multivisceral transplantation, aiming to resume enteral nutrition [6]. Intestinal Transplantation (IT) can be done isolated or combined with the liver or other organs (multivisceral), depending on the patient's clinical condition [7]. Patients who need to interrupt PN due to loss of central venous access or developed life-threatening complications related to the procedure have IT as the last life-saving therapy.

Due to the limited number of procedures and scarcity of centers that perform this type of transplant (only 47 centers worldwide are currently active), the medical field are generally unaware of the indications for IT, their current outcomes, and recent advances [2]. For example, in Brazil, there were only 15 patients transplanted in the last decade [8]. In this context, it is essential to investigate the clinical evolution of patients with SBS and chronic IF who end up undergoing IT in the studied country to raise awareness of the condition and pave paths to facilitate access to the procedure. This study aims to describe the clinical course of subjects with SBS and chronic IF who underwent IT in Brazil and their demographical and clinical characteristics.

## Materials and methods

### Study design and participants

This retrospective observational study was designed in accordance with STROBE guidelines for reporting observational studies [9]. All SBS-IF patients who underwent IT in two reference centers: Hospital das Clinicas da Faculdade de Medicina da Universidade de Sao Paulo where 5 patients were included, and Hospital Israelita Allbert Einstein where 2 patients were included (authorized by the Brazilian Ministry of Health to perform intestinal or multivisceral transplantation) from April 2011 to December 2021. The Informed Consent Form (ICF) was collected from all participants (ICF waiver was requested for deceased patients and for patients with loss of follow-up). The study protocol was reviewed and approved by both local research ethics committees.

### Data collection

Data were extracted from the patient's medical record and entered into an electronic case report form to capture information relevant to the study. Demographic and clinical characteristics data was collected up to 10 years before the IT. The patients’ course timeline was segregated into four periods: 1) From underlying condition to SBS-IF diagnosis, 2) From SBS-IF diagnosis to IT indication, 3) From IT indication to IT procedure, and 4) Overall (time from underlying condition up to IT procedure).

### Analyzed outcomes

The study's primary outcome was the SBS-IF patient's clinical course according to the four pre-defined milestones described above. The secondary outcomes were the description of clinical characteristics of underlying conditions and the clinical condition related to the development of SBS-IF, features and complications arising from the PN, reasons and characteristics of the IT, and clinical factors (anthropometric parameters, concomitant medication, and comorbidities) of the study population. The healthcare resource utilization in inpatient and outpatient settings was assessed.

### Study variables

For the primary outcome, the SBS-IF patient's clinical course was described in months as time from baseline to SBS-IF diagnosis, time from SBS-IF diagnosis to transplant indication, and time from IT indication to date in which the procedure was performed.

Demographic characteristics included gender, race/ethnicity, age at SBS-IS diagnosis and IT procedure, and patients who changed their city and state of origin because of the treatment. The underlying condition, the reason for the transplant indication, and the IT characteristics were assessed and described by categorical variables (absolute numbers and percentages). Patients could present more than one reason for transplant indication.

Body Mass Index (BMI) was calculated at SBS-IF diagnosis and IT indication period. Changes in the BMI category were also assessed. The classification of BMI was based on the Centers for Disease Control and Prevention's BMI definition [10].

The total number and percentage of complications of patients with the complication and the ratio of complications per patient by type were assessed. Data regarding overall types of SBS-IF complications and PN complications per patient, number of intestinal resections per patient and the length of remaining bowel (cm) and length of remaining colon (cm) in the last resection were also evaluated. The absolute number and percentage of patients according to the anatomy of the remaining bowel were assessed and expressed according to the SBS type classification [11].

Healthcare Resource Utilization (HCRU) was segregated by inpatient and outpatient settings. Inpatient procedures included a number of hospitalizations, length of stay and reasons for hospitalization. The number of hospital admissions from site admission up to IT was described as the number of hospitalizations per patient and per year. The reason for hospitalization and number of patients according to the number of hospital admissions were expressed by absolute number and percentage of the total hospitalizations. Outpatient and inpatient HCRU included the total number of procedures per patient over the study period.

Concomitant medications were described as the total number of unique patients that had used the medication at least once from the underlying condition up to IT. Concomitant medications were assessed according to the indication (SBS-IF management or related to a complication) and type of treatment (non-continuous [<6 months] or continuous use [>6-months]) and expressed by the absolute number and percentage of concomitant medication used. PN characteristics were assessed quantitatively and qualitatively between SBS-IF diagnosis and IT. Volume (mL/day [The monthly calculation for PN volume involves dividing the daily PN volume by the number of days during which PN was administered in a given month]), frequency (day/week), and type (ready-to-use or individualized) of PN, as well as the PN complication, were described over time.

### Statistical analysis

The primary and secondary outcomes were summarized using descriptive statistics, including several non-missing observations, mean, Standard Deviation (SD), Interquartile Ranges (IQR) and 95 % Confidence Interval (95 % CI) for the mean, where applicable. Categorical variables were described by simple and cross-contingency tabulation, with absolute frequencies and percentages with 95 % CI and percentages. All analysis, computations, and generation of tables, listings, graphics, and data for figures were performed using Python® version 3.6.9.

## Results

### Participants demographical and clinical characteristics

Of the 7 participants, all were male. Six (86 %) were self-declared as white, and 1 as black (14 %). The mean (SD) age of participants at SBS-IF diagnosis was 24.4 (13.4) years, and the mean age of the participants at IT was 27.7 (14.0) years (Table 1). Two participants had comorbidities at baseline: one subject with sigmoid volvulus surgery and another with depression (50 %). The most frequent underlying conditions were surgical complications, followed by intestinal volvulus and incisional hernia, corresponding to 4 patients (57 %), 2 patients (29 %), and 1 patient (14 %) with this underlying condition. PN-associated hepatic disease (57 %), loss of central venous (29 %), catheter-associated infections (14 %), and extensive/severe mesenteric-portal thrombosis were the reasons described for IT indication. The main type of IT performed was intestine only (6 participants). Only one subject underwent a multivisceral transplant, and the organs transplanted were intestine, liver, pancreas, and stomach (Table 1).

**Table 1:** Demographic, clinical, underlying conditions and intestinal transplant characteristics of eligible patients.

	All patients
**Age at diagnosis (years)**^a^	
Mean (SD)	24.4 (13.4)
Median (IQR)	20.0 (15.0‒32.0)
Min ‒ Max	13.0‒44.0
**Patient's age at transplant (years)**^a^	
Mean (SD)	27.7 (14.0)
Median (IQR)	23.0 (18.0‒35.5)
Min ‒ Max	14.0‒50.0
**Gender ‒ n (%)**^a^	
Male	7 (100 %)
Female	0 (0 %)
**Race/ Ethnicity – n (%)**^a^	
White	6 (86 %)
Black	1 (14 %)
**Patients that changed the city because of the disease, n (%)**	5 (71 %)
**Underlying condition, n (%)**^a^	
Surgical complications	4 (57 %)
Intestinal volvulus	2 (29 %)
Other (incisional hernia)	1 (14 %)
**Average number of underlying conditions per patient**	
Mean (SD)	1.1 (0.4)
Median (IQR)	1.0 (1.0‒1.0)
Min ‒ Max	1.0‒2.0
**Reason for transplant indication ‒ n (%)**	
Loss of central venous (sites)	2 (29 %)
Catheter-associated infections	1 (14 %)
Hepatic disease associated with TPN	4 (57 %)
Extensive/Severe mesenteric-portal thrombosis	1 (14 %)
Malnutrition	1 (14 %)
**Type of intestinal transplant ‒ n (%)**^b^	
Intestine only	6 (86 %)
Multivisceral transplantation (intestine, liver, pancreas and stomach)	1 (14 %)

Data are shown as mean (SD), median (IQR), minimum and maximum, total number (%), as appropriated. IQR, Interquartile Range; SD, Standard Deviation, TPN, Total Parenteral Nutrition.

a No missing, unknown, or not reported data.

b Patient could present more than one transplanted organ at once.

### Clinical course of patients with SBS and chronic IF up to transplantation hospitalizations related to SBS-IF

**Fig. 1:**
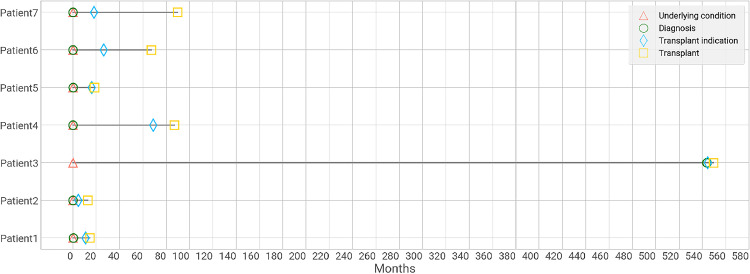
Clinical course description of SBS-IF patient's timeline from the date of underlying condition to the date of intestinal transplantation per patient (months).

The median time (IQR) from diagnosis of SBS-IF to the transplant indication was 15.7 (7.5‒22.1) months, between indication and IT was 8.1 (4.5‒29.7) months, and from underlying condition to IT was 67.3 (16.5‒88.5) months (Table 2). All patients had <1 month between the underlying disease and the SBS-IF diagnosis, except for one outlier patient with 544 months from the underlying condition to the SBS-IF diagnosis. The longest period between the SBS-IF diagnosis and the transplant indication was 69 months, and the shortest time was <1 month (Fig. 1). Between the indication of transplantation and IT, the longest time was 72 months, and the shortest time was 2 months. The total time (from underlying condition to IT) ranged from 13 to 550 months.

**Table 2:** Clinical course of SBS-IF patient's timeline up to intestinal transplantation (months) and hospitalizations related to SBS-IF disease since site admission until intestinal transplant.

	All patients
**Underlying condition up to SBS-IF diagnosis – Months**^a^	
Mean (SD)	77.8 (205.5)
Median (IQR)	0.0 (0.0‒0.3)
Min ‒ Max	0.0‒543.81
**SBS-IF diagnosis up to transplant indication – Months**^a^	
Mean (SD)	20.6 (22.9)
Median (IQR)	15.7 (7.5‒22.1)
Min ‒ Max	0.66‒68.7
**Transplant indication up to intestinal transplant – Months**	
Mean (SD)	21.5 (26.0)
Median (IQR)	8.1 (4.5‒29.7)
Min ‒ Max	2.46‒71.95
**Overall time (from underlying condition up to intestinal transplant) – Months**^a^	
Mean (SD)	119.9 (192.4)
Median (IQR)	67.3 (16.5‒88.5)
Min ‒ Max	12.65‒549.5
**Mean hospitalizations (per patient)**^a^	
Mean (SD)	4.3 (3.4)
Median (IQI)	4.0 (1.5‒6.0)
Min ‒ Max	1.0‒10.0
**Mean hospitalizations PPPY**^a^	
Mean (SD)	0.5 (0.3)
Median (IQI)	0.5 (0.3‒0.8)
Min ‒ Max	0.19‒1.01
**Number of patients hospitalizations per patient, N**	
1 hospitalization	2
2 hospitalizations	1
3 hospitalizations	0
4 hospitalizations	1
5 hospitalizations or more	3
**Reason for hospitalization** ‒ **n (%)**	
SBS-IF management	11 (37 %)
SBS-IF related complication management	7 (23 %)
Parenteral nutrition related complication	12 (40 %)

Data are shown as mean (SD), median (IQR), minimum and maximum, total number (%), as appropriated. IQR, Interquartile Range; SD, Standard Deviation, PPPY, Per Patient Per Year. SBS-IF, Short Bowel Syndrome Intestinal Failure.

a No missing data.

Of the 7 patients included in the study, two were hospitalized once, one was hospitalized twice, one was hospitalized four times, and three were hospitalized five times or more. The mean (SD) number of hospitalizations per patient was 4.3 (3.4), and the mean (SD) number of hospitalizations per patient per year was 0.5 (0.3). The most common reason for hospitalization was PN-related complications (40 %), followed by SBS-IF management (37 %) and SBS-IF-related complications (23 %) (Table 2).

### Parenteral nutrition performed by the patient and anthropometric characteristics

Table 3 describes the parenteral characteristics of SBS-IF patients. The mean (SD) volume of PN from diagnosis of SBS-IF to bowel transplantation was 1502.4 (238.9) mL/day (for ready-to-use PN type) and 2040.0 (432.6) mL/day (for individualized PN type). The median duration of PN was 64.1 (IQR 12.0‒66.0) months for ready-to-use PN and 9.0 (IQR 3.5‒13.0) months for individualized PN.

**Table 3:** Parenteral nutrition performed by the patient and anthropometric characteristics between SBS-IF diagnosis up to intestinal transplantation.

	Total of parenteral nutrition	Type of parenteral nutrition	
		Ready to use (Outpatient)	Individualized (Inpatient)
**Mean volume of PN (mL/day)**^a^			
Mean (SD)	1608.3 (357.8)	1502.4 (238.9)	2040.0 (432.6)
Median (IQR)	1500.0 (1400.0–1743.0)	1400.0 (1400.0–1667.0)	2000.0 (1900.0–2474.0)
Min ‒ Max	1000.0–2729.0	1000.0–2150.0	1000.0–2729.0
Missing data	0	0	0
**Duration of PN (month)**^a^			
Mean (SD)	23.5 (27.1)	43.8 (32.5)	9.0 (7.3)
Median (IQR)	12.0 (4.8‒31.7)	64.1 (12.0‒66.0)	9.0 (3.5‒13.0)
Min ‒ Max	0.0‒72.02	5.03‒72.02	0.0‒20.96

**BMI (Kg/m^2^) ‒ n (%)**		**Event time**	
		**At SBS-IF diagnosis**	**Previous to transplant**
Normal		5 (71 %)	4 (57 %)
Mild underweight		0	1 (14 %)
Moderate underweight		0	2 (29 %)
Severe underweight		2 (29 %)	0
**Changes in BMI category between SBS-IF diagnose and intestinal transplant**^b^		**All patients**	
No change		4 (57 %)	
Increase		2 (28 %)	
Decrease		1 (14 %)	

Data are shown as mean (SD), median (IQR), minimum and maximum, total number (%), as appropriated.

IQR, Interquartile Range; SD, Standard Deviation; PN, Parenteral Nutrition, BMI, Body Mass Index; cm, centimeters; Kg, Kilograms; m^2^, Square meter; mL, Milliliters; SBS-IF, Short Bowel Syndrome Intestine Failure.

Duration of parenteral nutritional (month): considered the total amount of time of all PN.

a No missing data.

b BMI values changes throughout the study period.

At diagnosis of SBS-IF, five patients had normal BMI, and two were severely underweight. Before IT, four patients had a normal BMI; 1 had mild underweight, and 2 had severe underweight. For those patients, only three changed the BMI category between the SBS-IF diagnosis and IT: two patients increased the BMI category from severe underweight to moderate underweight or mild underweight, and one patient decreased the BMI category from normal to moderate underweight (Table 3).

### Description of SBS-IF and PN complications characteristics and anatomy site and length of the remaining bowel

A total of 12 SBS-IF complications were reported, of which malnutrition (50 %) was the most frequent, followed by liver disease associated with intestinal failure (17 %), bone disease (8 %), infection by *Staphylococcus Aureus* (8 %), liver disease (8 %) and osteoporosis (8 %). The mean (SD) number of SBS-IF complications per patient was 2.4 (2.2), and PN-related complications were 3.86 (2.19) per patient (Table 4).

**Table 4:** Description of SBS-IF and parenteral nutrition complications characteristics and anatomy site and length of the remaining bowel.

	All patients	Patients with complication, n (%)	Ratio of complications per patient^b^
**Type of SBS-IF complication ‒ n (%)**			
Bone Disease	1 (8 %)	1 (20 %)	1
Malnutrition	6 (50 %)	2 (40 %)	3
Infection by staphylococcus aureus	1 (8 %)	1 (20 %)	1
Intestinal failure associated liver disease	2 (17 %)	2 (40 %)	1
Liver disease	1 (8 %)	1 (20 %)	1
Osteoporosis	1 (8 %)	1 (20 %)	1
**Average SBS-IF complication per patient**^a^			
Mean (SD)	2.4 (2.2)	‒	‒
Median (IQR)	1.0 (1.0‒3.0)	‒	‒
Min ‒ Max	1.0‒6.0	‒	‒
**Average PN complication per patient**^a^			
Mean (SD)	3.86 (2.19)	‒	‒
Median (IQR)	3.0 (3.0–4.5)	‒	‒
Min - Max	1.0–8.0	‒	‒
**Anatomy of remaining bowel - anatomic classification – n (%)**			
Jejunostomy/ileostomy ‒ SBS Type 1	2 (29 %)	‒	‒
Jejunocolic anastomosis ‒ SBS Type 2	4 (57 %)	‒	‒
Jejunoileal anastomosis	1 (14 %)	‒	‒
**Resections characteristics**			
Mean (SD)	1.3 (0.5)	‒	‒
Median (IQR)	1.0 (1.0‒1.5)	‒	‒
Min ‒ Max	1.0–2.0	‒	‒
**Length of remaining bowel (cm)**^c^			
Mean (SD)	46.7 (71.7)	‒	‒
Median (IQR)	15.0 (10.0‒40.0)	‒	‒
Min ‒ Max	6.0‒206.0	‒	‒
**Length of remaining colon (cm)**^c^			
Mean (SD)	40.0 (31.1)	‒	‒
Median (IQR)	40.0 (20.0‒60.0)	‒	‒
Min ‒ Max	0.0‒80.0	‒	‒

Data are shown as mean (SD), median (IQR), minimum and maximum, total number (%), as appropriated.

IQR, Interquartile Range; SD, Standard Deviation, cm, centimeter; SBS, Short Bowel Syndrome.

a No missing data.

b Ratio of complication per type of SBS-IF complication.

c In the last resection.

Considering the intestinal resection, a mean (SD) of 1.3 (0.5) was performed per patient, and the location of the remaining intestinal anatomy was jejunocolic anastomoses, jejunostomy/ileostomy, jejunoileal anastomosis, duodenocolic anastomosis, duodenostomy, and total colectomy. The entire length of the remaining bowel (at the last resection) of all patients ranged from 6 to 206 cm with a mean (SD) and median (IQR) length of the remaining intestine of 46.7 (71.7) and 15.0 (10.0‒40.0) cm, respectively. The total length of the remaining colon (at the last resection) of all patients ranged from 0 to 80 cm, with a mean (SD) and median (IQR) length of the remaining colon of 40 (31.1) and 40.0 (20.0‒60.0) cm, respectively (Table 4).

### Procedures performed in inpatient and outpatient settings

A total of 5536 inpatient procedures and 6621 outpatient procedures were performed. The assessment of liver function was the most frequent, with 1635 tests performed in the inpatient setting and 1996 tests performed in an outpatient setting. Kidney function was the second most performed procedure in inpatient setting (1521) and outpatient setting (1827). The hemogram was the third most performed procedure in inpatient settings (922) and outpatient settings (1126). The number (mean [SD] per patient) of laboratory tests performed in the hospital ranged from 233.57 (179.5) for liver function to 1.86 (2.61) for fecal examination. The mean number of tests performed in outpatient settings ranged from 332.7 (278.8) for liver function to 1.8 (2.9) for fecal examination. A total of 60 imaging exams were conducted in hospital and 53 imaging exams in outpatient setting. The three most performed in-hospital imaging exams (mean [SD] per patient) were abdominal X-rays (1.57 [1.4]), abdominal ultrasound (1.43 [1.62]) and endoscopy (0.86 [1.21]) and outpatient was abdominal radiography (2.5 [1.9]), computed tomography (2, 3 [1.6]) and abdominal ultrasound (2.0 [2.1]). The total number of exams performed in-hospital was 60, and 53 in the outpatient setting. A total of 10 surgeries and four biopsies were performed in the hospital setting Table 5).

**Table 5:** Procedures performed in inpatient and outpatient setting.

		n (%)^a^		
**Indication of concomitant medication ‒ n (%)**^b^		149		
SBS-IF management		90 (60 %)		
SBS-IF related complications		59 (40 %)		
**Treatment type ‒ n (%)**^b^		149		
Non-continuous use (occasional/sporadic)^c^		96 (64 %)		
Continuous use^d^		53 (36 %)		

	**Inpatient procedures performed**	**Outpatient procedures performed**		
	**Total number of procedures**	**Mean (SD)**^a^	**Total number of procedures**	**Mean (SD)**^a^
**Lab test**				
Liver function assessment	1635	233.57 (179.5)	1996	332.7 (278.8)
Renal function assessment	1521	217.29 (168.35)	1827	304.5 (262.9)
Coagulation Profile	858	122.57 (92.08)	810	135.0 (91.1)
Hemogram	922	131.71 (120.01)	1126	187.7 (178.5)
Micronutrients parameters	587	83.86 (59.11)	851	141.8 (120.0)
Fecal tests	13	1.86 (2.61)	11	1.8 (2.9)
**Total**	5536	‒	6621	‒
**Imaging exams (total number of procedures)**				
Endoscopy	6	0.86 (1.21)	5	0.8 (1.0)
Colonoscopy	2	0.29 (0.49)	4	0.7 (1.2)
Abdominal X-Rays	11	1.57 (1.4)	15	2.5 (1.9)
Abdominal ultrasound	10	1.43 (1.62)	12	2.0 (2.1)
MRI report	1	0.14 (0.38)	0	0.0 (0.0)
Computed Tomography (CT)	20	2.86 (2.34)	14	2.3 (1.6)
Liver biopsy	5	0.71 (0.49)	2	0.3 (0.8)
GI biopsy	5	0.71 (0.95)	1	0.2 (0.4)
**Total**	60	‒	53	‒
**Total of surgery**	10	‒	‒	‒
**Total of biopsy**	4	‒	‒	‒

Data are shown as mean (SD) and total number (%), as appropriated.

SD, Standard Deviation; SBS-IF, Short Bowel Syndrome Intestine Failure; GI, Gastro-Intestinal.

* The same patient might have used more than one concomitant medication, and the same medication might have been used more than once.

a Average per patient for the entire study period.

b Number of events (records of concomitant medication use).

c It is considered the use of medication, occasionally or dispersedly.

d It is considered the permanent use of a medication for a period greater than or equal to 6 months. Usage can be daily or at defined fixed intervals.

In total, 149 concomitant medications were reported during the study period, of which 60 % were for managing SBS-IF and 40 % were for managing complications related to SBS-IF. Most of the treatments 96 (64 %) were non-continuous, and 53 (36 %) were continuous (Table 5). Antibiotics and anticoagulants were the most used concomitant medications during the study period, with all patients having a record of at least one antibiotic and one anticoagulant drug (100 %) use. Six patients used vitamins, minerals, carbonic acid, and supplements at least once (86 %). Antifungal, proton pump inhibitor, antidiarrheal, and trace elements were used at least once by five patients (71 %), and bile acid class medication by four patients (57 %).

All 7 patients included in the study were followed up until June 30, 2024. As of this date, all patients remain alive.

## Discussion

According to the International Intestine Transplant Registry (ITR), growth and improvement in graft survival rates over the last two decades have been reported [12]. It remains the ultimate treatment for patients with irreversible IF who develop life-threatening complications associated with PN. The busiest transplant centers are in North America and Europe, but the greatest percentage growth in activity has occurred in South America in the last few years [12]. This study collected up to 10 years of medical history of 7 eligible patients who underwent IT in Brazil. To the best of our knowledge, this is the first Brazilian real-world study on the clinical course of transplanted SBS-IF patients. It revealed the timeframe among the disease phases up to IT and the clinical characteristics of this population.

SBS is a rare malabsorptive disorder as a result of the loss of bowel mass, mostly secondary to surgical resection of the small intestine, followed by vascular diseases, neoplasms, or inflammatory bowel disease [13]. In the present study, the most common reason was surgical complications. The spectrum of the disease is widely variable from single micronutrient malabsorption to complete intestinal failure, depending on the remaining length of the small intestine, the anatomical portion of the intestine, and the function of the remnant bowel [14]. Worldwide studies that assessed the general characteristics of patients who underwent IT found a predominance of males [[15], [16], [17]], similar results as the authors described in the present study where all included patients were young men at SBS-IF diagnosis and IT.

There are a vast number of reasons that can cause SBS-IF disease, and they can directly impact the timeframe and journey of the patients to diagnosis and treatment. Mild to moderate chronic conditions could take long periods up to SBS-IF development, while severe and acute conditions could take short periods up to disease. Intestinal obstruction, intestinal ischemia, abdominal trauma, and mesenteric infarction are examples of acute conditions that may need IT more quickly, as the damage to the intestine is more severe, and it may not be possible to manage the disease with specific treatments. On the other hand, chronic diseases such as Crohn's disease, gastroschisis, and necrotizing enterocolitis may have a slower disease course. They can be managed with medical therapies and changes in the patient's diet for a prolonged period, requiring a long time between treatment and IT [18].

SBS may result in intestinal resection, diarrhea, dehydration, weight loss, and nutritional deficiencies, requiring PN or intravenous fluids and some patients with SBS-IF who require long-term PN have significant complications that end up having to undergo IT [19]. The main reasons for indicating IT in this study were hepatic disease associated with PN and loss of central venous and catheter issues, consistent with the literature [16,20]. The authors found a mean of 408.75 liver function assessments per patient and 380.25 renal function assessments per patient only during the hospitalizations. Despite advances in the management of SBS-IF in recent decades, this disease continues to carry a high burden of morbidity and mortality [21].

The clinical course between the underlying condition and the IT assessed in this study revealed a median time of 67 months, with the time between the SBS-IF diagnosis and the transplant indication being the longest (median of 16 months). It is important to highlight that if the authors considered the mean value found in the study, the highest period would be between the underlying condition and the SBS-IF diagnosis. However, this period was the shortest one if the authors considered the median value found in the study. This divergence could be explained by an exceptional case in which a patient contributed >205 months due to a chronic condition. The other patients spent less than one month at this stage of the clinical journey since the diseases responsible for developing SBS-IF were mostly acute, such as surgery complications.

IT is also a consideration in SBS patients who have failed more conservative approaches to treatment [22]. A classic review of the international experience with transplantation indicated high morbidity (50 %) but with a significant proportion of patients achieving nutritional autonomy (40 %). Hence, it is recommended that IT be reserved for those who have failed other treatment options and have developed life-threatening complications related to TPN. There are three main types of IT: the small bowel graft with or without the liver and a multivisceral transplantation including the intestine, liver, and stomach [23], the isolated small bowel graft being the most common [12]. According to the 2015 ITR, 2887 ITs have been performed worldwide since 1985, of which only 24 % were multivisceral transplants [15].

The present study identified a median of 8.1 months between indication for transplantation and IT. In agreement, a study conducted in Latin America identified that the mean waiting time between the indication and IT was approximately 5.4 and 7.5 months [8], as another study that analyzed 88 patients between 1991 and 2010 reported that the mean time between the indication and the performance of the transplant was approximately 5 to 7 months [24].

One of the major study findings was the substantial average number of hospitalizations was 4.3 admissions per patient and 0.5 admissions per patient per year with a mean and median length of stay of 50 and 16 days, respectively. The main reason for hospitalization was complications related to PN (40 %), followed by SBS-IF management (37 %) and its complications (23 %). These findings align with a study of 331 patients with SBS-IF receiving PN that found that hospital admissions were 2.5 per year, the average length of stay was 7 days, and complications related to the PN catheter were the most frequent causes of hospitalizations [17]. In a Portuguese cohort, SBS-IF-related hospitalizations were notoriously frequent; an annual average per patient of two admissions and 31 days of patient's length of stay was estimated, which in practical terms translates into one month of hospitalization per year. The same findings were reported by a retrospective cohort study in Danish SBS-IF patients over a 46-year study period, which estimated an admission incidence of 2.5 episodes per year [17].

The significant reduction of the intestinal surface and disturbances in the production of bile acids and digestive enzymes in patients with SBS affect the absorption of essential nutrients [25]. Therefore, to address the significant reduction in the intestinal surface and the absorption of nutrients, it is expected to find a high number of vitamins, minerals, carbonic acid, and supplements by patients, which are essential to maintaining the patient's nutrition in the long term. The results of this study showed that the concomitant medications most used by patients along the journey were antibiotics (meropenem [100 %] and vancomycin [86 %]), followed multivitamin (86 %), antifungals (fluconazole (Diflucan) [71 %]), anticoagulants (heparin [71 %] and enoxaparin [71 %]) and omeprazole (71 %). SBS patients may have a higher risk of intestinal infections for several reasons, such as decreased immunity due to bowel malfunction [26], urinary tract infections [25] and PN administration, which may increase the risk of catheter-related infections [1]. Patients with SBS often have alterations in the intestinal microbiota, including an imbalance between bacteria and fungi, which can increase the number of pathogenic fungi [27]. This is why antifungals appear as the second most used by patients in the study and antibiotics as the first.

PN, which is an indicated alternative for the treatment of SBS-IF patients, requires inpatient or outpatient coverage and can also represent a significant economic burden for patients. For example, Medicare in the United States only covers 80 % of PN costs and only if specific conditions are met [19]. Although potentially lifesaving, PN is expensive, invasive, and associated with numerous complications and harmful effects on health and quality of life. The present study showed that patients' mean duration of PN was 44 months. A survey of 124 adult patients with SBS who had permanent intestinal failure showed that the duration of PN was also 44 months (range 1 to 160 months). This same study also suggests that there is an adaptation of the functions of the intestine that begins after 1 to 3 years in adults, which tends to decrease dependence on PN after this period [28]. However, the duration, volume, and frequency of PN in SBS-IF patients may vary according to the clinical status of the patients and the individual response of each patient, being a very individualized treatment, making it difficult to compare the results of this study with the literature.

A study that analyzed patients with SBS who used PN over two years showed that patients did not have large significant differences in BMI. These findings were similar to those found in the present study, in which only three patients changed their BMI classification between the diagnosis of SBS-IF and the IT: one patient went from normal BMI to moderate underweight, one patient went from severe underweight to moderately underweight and another went from severe underweight to mild underweight [29]. These findings align with other studies in the literature, which indicate that PN can satisfactorily control the BMI of patients within the recommended limit [30].

Limitations are present in this type of retrospective nature of the study design and chart review data collection. The data are limited by the information reported and may have some missing or incomplete data, although data clearance was undertaken to maximize these errors. The utilization of the data from two reference centers could generate selection bias. Due to the absence of a centralized national patient registry, data validity in this retrospective analysis depended on accurate medical records. Although the study sample is small, the rarity of this disease on a global scale should be accounted for.

## Conclusions

By analyzing ten years of data, this study was able to characterize the profile of patients with SBS-IF who underwent IT surgery in Brazil. This study was able to understand data gaps regarding patients with SBS-IF undergoing IT surgery in Brazil.

The findings of the present study can be useful to better understand the journey of patients with SBS-IF until IT, providing real-world evidence that can help in the elaboration of health policy guidelines and improve the quality of life of these patients.

## Declaration of competing interest

The authors declare the following conflicts of interest: YLB; MHMR; LH; RAAP: ADWL: APCSB; IC; GGR; FM; and LACA: Has acted as speaker in Takeda Distribuidora Ltda in medical-scope events. CYS and BF are employees of Takeda Distribuidora Ltda, Brazil.
